# Modulation of motor inhibition by subthalamic stimulation in obsessive-compulsive disorder

**DOI:** 10.1038/tp.2016.192

**Published:** 2016-10-18

**Authors:** A Kibleur, G Gras-Combe, D Benis, J Bastin, T Bougerol, S Chabardès, M Polosan, O David

**Affiliations:** 1 Université Grenoble Alpes, Grenoble, France; 2 Inserm, U1216, Grenoble Institut des Neurosciences, Grenoble, France; 3 Clinique Universitaire de Psychiatrie, Pôle Psychiatrie Neurologie, Centre Hospitalier Universitaire, Grenoble, France; 4 Clinique Universitaire de Neurochirurgie, Pôle Tête et Cou, Centre Hospitalier Universitaire, Grenoble, France

## Abstract

High-frequency deep brain stimulation of the subthalamic nucleus can be used to treat severe obsessive-compulsive disorders that are refractory to conventional treatments. The mechanisms of action of this approach possibly rely on the modulation of associative-limbic subcortical–cortical loops, but remain to be fully elucidated. Here in 12 patients, we report the effects of high-frequency stimulation of the subthalamic nucleus on behavior, and on electroencephalographic responses and inferred effective connectivity during motor inhibition processes involved in the stop signal task. First, we found that patients were faster to respond and had slower motor inhibition processes when stimulated. Second, the subthalamic stimulation modulated the amplitude and delayed inhibition-related electroencephalographic responses. The power of reconstructed cortical current densities decreased in the stimulation condition in a parietal–frontal network including cortical regions of the inhibition network such as the superior parts of the inferior frontal gyri and the dorsolateral prefrontal cortex. Finally, dynamic causal modeling revealed that the subthalamic stimulation was more likely to modulate efferent connections from the basal ganglia, modeled as a hidden source, to the cortex. The connection from the basal ganglia to the right inferior frontal gyrus was significantly decreased by subthalamic stimulation. Beyond motor inhibition, our study thus strongly suggests that the mechanisms of action of high-frequency subthalamic stimulation are not restricted to the subthalamic nucleus, but also involve the modulation of distributed subcortical–cortical networks.

## Introduction

The physiopathology of obsessive-compulsive disorder (OCD) is related to dysfunctions of cortical–subcortical loops,^1, 2, 3^ including the ventral cognitive circuit partly composed of the anterolateral orbitofrontal cortex involved in motor and response inhibition.^3, 4^ More specifically, for this study, the subthalamic nucleus (STN), an interesting target for deep brain stimulation (DBS) therapy in OCD,^5, 6^ was shown to be a key node of the cortico-striato-thalamic-cortical loop of motor inhibition.^7^ It receives direct afferences from prefrontal regions^8^ involved in executive functions, such as set-shifting^9^ and decision-making,^10^ that are impaired in OCD.

It has been shown that patients with OCD may show response inhibition impairments, in relation to both motor and cognitive inhibition.^11, 12^ However, specifically on motor inhibition, the literature shows heterogeneous results, as no significant difference in response inhibition performances could be detected between OCD patients and healthy controls in several studies.^13, 14, 15^ Operationally, the inhibition performance can be evaluated in patients using the stop signal task (SST).^16^ STN-DBS was also shown to impair inhibition and increase impulsivity in Parkinson disease using a Go/NoGo task.^17^ However, STN-DBS effects on performances during the SST are still controversial, with studies reporting improved inhibition,^18^ altered inhibition^19^ and altered executive control.^20^ Overall, STN-DBS was shown to increase impulsivity in high-conflict situations, which could be due to the modulation of STN and medial prefrontal cortex interactions that influence the reaction time in decision-making.^21^ Because the STN is supposed to act as a brake in high-conflict context by delaying the thalamic outputs onto motor areas before taking the right decision, this brake could be perturbed under STN-DBS, leading to more impulsive reactions, premature responses and/or shorter reaction times.^22, 23, 24^

Inhibition processes involved in the SST are supposed to activate at least two brain pathways. The first one underlies fast reactive inhibition. It connects the right inferior frontal cortex to the STN^25^ that sends outputs towards the subtantia nigra pars reticulata and the globus pallidus internal segment, which in turn act on the thalamus to inhibit the motor output. The second process is supposed to be slower and to block motor outputs afterwards.^26^ It involves the dorsolateral prefrontal cortex (DLPFC), the pre-supplementary motor area (preSMA), which modulates the STN activity through hyperdirect projections, and the dorsal anterior cingulate cortex (dACC). The dACC probably monitors the conflict between Stop and Go processes, and reinforces the gating at the striatum level to block Go processes. Finally, another inhibition network, the indirect pathway, may be activated before the stop signal cue to prepare inhibition (proactive inhibition) by pre-activating the STN and at the same time slowing responses in uncertain environments.^27^

Because the SST involves different types of inhibition, it is a relevant framework to study the effects of STN-DBS on inhibition cortical–subcortical pathways. It is more specific on inhibition that the Go/NoGo task that involves an additional switch process. One report in Parkinsonian patients^28^ studied inhibition processes with the SST and STN-DBS by suggesting that the slow hyperdirect pathway could be the most modulated because the DLPFC and the dACC showed a decreased metabolism with STN-DBS. Because STN-DBS effects during the SST are probably different in Parkinsonian patients and OCD patients, we revisit here the neurobiological underpinnings of STN-DBS modulation of motor inhibition with improved temporal precision, and in the absence of dopaminergic depletion, by studying brain networks involved in the SST from electroencephalographic recordings (EEG) in a cohort of patients suffering from severe refractory OCD.

## Materials and methods

### Patients

The 12 patients (8 females and, 4 males; 42±8 years old) included in this study were all right handed and had normal or corrected to normal vision. They were recruited from the Grenoble University Hospital. Before surgery, average disease duration was 18±9.2 years, and Yale-Brown Obsessive Compulsive Scale (YBOCS) score was 34.3±3.2. At the time of the study, YBOCS baseline score was 20±9.1 with a clinical improvement of 41±28%. Table 1 summarises clinical and demographic information. No recorded data were excluded from the analysis.

### Deep brain stimulation protocol

The patients have been treated for 38±19 months with bilateral STN-DBS according to a protocol already published.^5, 6^ The patients were implanted bilaterally with two electrodes 3389 connected to a Kinetra stimulator (Medtronic, Minneapolis, MN, USA), using a stereotactic surgical procedure aiming at targeting the ventral non-motor part of the STN (anterior by 2 mm from the middle of the anterior–posterior commissural line, 11 mm lateral to the midline and 3 mm below the anterior–posterior commissural line). The stimulation frequency (130 Hz) and pulse width (60 μs) were the same for all patients. Other parameters, such as stimulation amplitude and montage, were adjusted individually during several weeks or months to obtain the best possible clinical response.

The EEG research protocol was approved by the Ethics Committee of Grenoble University Hospital (N° ID RCB: 2012-A00490-43). All the patients volunteered to participate in the study, gave written informed consent and received no financial support. The patients performed the task with STN-DBS ON and STN-DBS OFF, in a randomized and double-blind order during 2 successive days. The patients waited for 12 h between the stimulation changes and the EEG recordings to allow some washout of DBS effects. Neither the patients nor the psychiatrist doing clinical evaluations (MP) knew about the stimulation setting during 2 days of the experiment.

### Stop signal task

**Figure 1:**
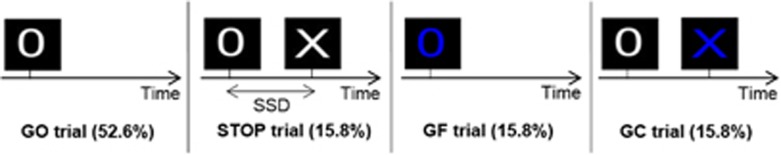
Four conditions of the stop signal task: Go, STOP, Go Fast (GF) and Go Certain (GC). SSD, stop signal delay.

We used a stop signal paradigm already developed and tested by our group postoperatively in patients with OCDs^29^ and patients with Parkinson disease.^27^ The task was composed of four types of trials allowing to investigate different forms of motor inhibition (Figure 1). The subjects were trained to perform the SST before starting EEG recordings. The recording session was decomposed into blocks of 15 min and 100 trials to allow the patients to rest. We used from two to four blocks for each patient depending on his/her motivation and clinical condition.

The stop signal delay (SSD), that is, the time interval between the presentation of the Go and Stop cues (Figure 1), was updated after each trial to make the performance converge to 50 percent of correct inhibition. The SSD was increased by 50 ms if the subject succeeded in inhibiting his/her movement, making the task more difficult in the following trial. If the patient failed to inhibit his/her movement, the SSD was decreased by 50 ms on the next trial. The initial SSD of the first block was chosen as the mean SSD of the training blocks. The initial SSD of each following block was set at the mean SSD values of the four last STOP trials of the previous block of task. To avoid unwanted strategies that could bias the estimation of the stop signal reaction time (SSRT: inhibition performance index), for example, waiting for the Stop cues, the subjects were asked to respond as quickly and accurately as possible to the Go cues. In addition, a low number of STOP trials (<16% of the trials), compared with the high number of GO trials (52.6% of the trials) was used. The SSRT was computed from the Go reaction time distribution and the SSD (Supplementary Materials).

### EEG recordings

We recorded EEG activity using 96 active electrodes (Acticap, Brain Products, Gliching, Germany) with a sampling rate of 2500 Hz and bandpass of acquisition between 0.01 Hz and 1200 Hz. Reference and ground were taken at FCz (midline, central frontal) and AFz (midline, anterior frontal), respectively. Two additional electrodes measured the vertical electrooculogram, and two other electrodes were placed on the neck close to the stimulation leads to measure the artifact of stimulation. The scalp electrode coordinates relative to three fiducials (nasion, left tragus and right tragus) were obtained from manual measurements using callipers following an *ad hoc* procedure derived from Koessler *et al.*^30^

### EEG pre-processing

EEG pre-processing was performed using EEGLAB software.^31^ The data were band-pass-filtered between 1 and 45 Hz to remove line noise and DBS artifacts, and then downsampled to 250 Hz. Bad channels were visually detected and removed from further analyses. Independent component analysis (ICA) on the EEG and electrooculogram channels allowed selecting and suppressing the components corresponding to the ocular and muscular artifacts.^32^ It was done in two successive ICA passes, by suppressing few components in each ICA pass, as we found ICA on pre-cleaned data from the first ICA was still efficient in removing remaining artifacts. The signals were then epoched and low-pass filtered with a cutoff at 30 Hz. The single trials were reviewed visually one by one to remove the trials that still showed significant artifacts. The cleaned EEG signals were then averaged using a common average reference and the Go signal correction (see below) was applied to estimate the inhibitory response to all four conditions (ON or OFF DBS, and successful stop (SS) or unsuccessful stop (US) trials) with the time origin at the presentation of the Stop cue.

### Estimation of motor inhibition ERPs

The estimation of motor inhibition event related potential (ERPs) was based on the horse race model,^33^ which considers that the Stop and Go processes are independent and that the first to reach its threshold (corresponding to a minimum limit of activity of Go or Stop neurons) wins the race and is executed. The time required to reach the threshold for the Go process is variable, as reflected in the variance of Go RTs, and thus motivates probabilistic methods to calculate the SSRT (Supplementary Materials). Similarly, the Go and Stop processes overlap during the STOP trials. To infer the EEG component directly due to the inhibition processes in responses to STOP trials, it is necessary to remove the components generated by the overlapping GO processes. We first used the signals from the GO trials to obtain an EEG signature of GO processes void of any ongoing reactive inhibition processes. Second, we applied a correction to the STOP trials that was different according to whether the trials were successful or not (Supplementary Materials).

For each patient and each STN-DBS condition (ON or OFF), we computed the amplitudes and latencies of the peak of the ERP on electrode F4, which showed the highest effect of DBS, and for the global field power (GFP, defined as the sum over electrodes of the absolute value of ERP minus scalp-wide average ERP), which gives a more global information on DBS modulation across the scalp. The peaks of the signals were detected using in-house Matlab codes for peak detection based on the Matlab ‘findpeaks' function.

### Source reconstruction

Source reconstruction was performed using SPM12 software (www.fil.ion.ucl.ac.uk/spm12). The EEG head model was computed for each patient with the boundary element method using the segmentation of patient's anatomical T1-weighted magnetic resonance imaging coregistered with measured EEG electrode coordinates. Intersubject registration was done by reporting source localization results in the Montreal Neurological Institute (MNI) space. The spatial resolution was set to ‘normal' (8196 cortical vertices). For each stimulation condition (OFF and ON stimulation), the sources of scalp data were estimated on the time interval (−200; 500) ms centered on the stop signal, using the minimum norm inversion method. We epoched the inverted data for each trial in small time windows of interest (100 ms duration) centered on the individual mean peak latencies between ON and OFF DBS for the SS trials, and during the baseline (−150 to −50 ms before stimuli presentations). For each trial, we subtracted the source activity in the baseline from the activity in the time window of interest and then we smoothed the resulting images from each trial (8 mm isotropic kernel). Finally, a full factorial design was performed across patients (with one covariate per patient) including the resulting images for each latency to detect the common source activation linked to inhibition processing between patients (using a statistical T threshold of *P*<0.005 uncorrected), and contrasts between ON and OFF stimulation SSs trials were used to study the significant effects of STN-DBS.

### Dynamic causal modeling

Dynamic causal modeling (DCM) was used to infer changes of long-range connectivity within the networks of inhibition that could explain changes of ERP amplitude observed between experimental conditions in SS trials.^34^ The inhibition ERPs were assumed to originate from different neuronal populations other than the ones responsible for the Go processes because the difference performed to correct for the Go process rests on the hypothesis that Go and Stop processes are independent (horse race model). This independence is supported by local recordings in the STN, which showed distinct neuronal population for motor response versus SSs in the SST.^29^ Under this assumption, the DCM method can be readily applied on a difference of ERPs, which was assumed to keep inhibitory processes only. The ERP used in DCM was low-pass filtered below 15 Hz to ease the optimization of DCM parameters. On the basis of the cortical sources identified previously and of the literature of motor inhibition, we selected six regions of interest that were defined according to their MNI coordinates {*X*;*Y*;*Z*}: the bilateral inferior frontal gyrus (IFG) {±54;12;18}, the bilateral DLPFC {±37;27;39} and the medial preSMA/dACC {0;−6;56}. To keep the model as simple as possible, we sacrificed the precision of the modeling of cortical/subcortical/cortical loops, and the basal ganglia (BG) structures involved in the inhibition networks were modeled as a single hidden source.

The motor inhibition process was triggered by a visual cue, the model input, which was assumed to target first the IFG and DLPFC. From there on, we modeled two inhibition pathways: (i) a fast pathway connecting the IFG and the BG directly; (ii) a slower pathway involving feedback interaction from the DLPFC to the preSMA, which projected to the BG to maintain the inhibitory processes in time.^26^ Cortico-cortical connections within the same hemisphere were bidirectional. Top-down and bottom-up processes were modeled using forward and backward connections. Interhemispheric connectivity between homologous regions, that is, IFG and DLPFC, used lateral connections. Because the deep source was a rough approximation of both input and output structures of the BG, we modeled cortical–subcortical–cortical interactions as hierarchically non-informative using bidirectional lateral connections.

The spatial model chosen for the electromagnetic sources was equivalent current dipole (ECD model in SPM12). For each patient, the DCM parameters were estimated on the ERP of the two experimental conditions (ON and OFF stimulation) at the same time. Extrinsic connections involving the BG were modulated between experimental conditions to explain the condition-specific differences of the ERP. We built nine different models to test whether the STN-DBS would specifically modulate in this task connections with either the IFG or the preSMA/dACC, or if both cortical areas were modulated by stimulation. The nine models were grouped into three families: one family testing whether STN-DBS could modulate effective connectivity of IFG only, another testing the modulation of effective connectivity of medial frontal areas only and finally a family testing whether DBS could induce the modulation of both regions. Intrinsic connections remained stationary between conditions.

ERPs were fitted from 0 to 500 ms, with Hanning windowing and nine spatial modes were used to reduce the dimensionality of the data. To minimize the effect of local minima of the negative free energy when optimizing DCM parameters, we run nine estimations of each model for each patient with different initial parameters and kept the solution with the best fit. We performed Bayesian model selection with random effects^35^ on the resulting posterior probabilities of the nine models grouped on three families to determine the model and the family with the highest exceedance probability. We performed a Bayesian model averaging on the most probable family and compared the posterior connections strengths from this analysis with paired *t*-test. Finally, we computed the source activity from the most probable family at the six DCM nodes by averaging on the three models of this family across patients the corresponding time courses (normalized by their s.d. across conditions).

### Code availability

Data were processed using open source software: SPM12 (Wellcome Trust Centre for Neuroimaging, UK) and EEGLAB (Swartz Center for Computational Neuroscience, USA).

## Results

### Behavior

The change in YBOCS introduced by the change in STN-DBS was close to significance with similar effect between the obsession and compulsion subscales (on average: 22.17 with STN-DBS OFF—10.92 in the obsession subscale and 11.25 in the compulsion subscale; 19.25 with STN-DBS ON—9.33 in the obsession subscale and 9.92 in the compulsion subscale; Wilcoxon signed-rank test: *P*=0.0542). The patients performed the task well: The mean task error rate was 3.7±7.9% (mean±s.e.m.) and the success rate in the STOP trials was 49.4±8.9%. Under STN-DBS ON, the patients had shorter reaction time in Go trials (GORT OFF: 649±61 ms; GORT ON: 599±41 ms; *P*=0.078 uncorrected paired *t*-test), GC trials (GCRT OFF: 708±60 ms; GCRT ON: 655±39 ms; *P*=0.104 uncorrected paired *t*-test) and GF trials (corresponding to reaction times to GO signals never followed by a Stop signal; GFRT OFF: 565±47 ms; GFRT ON: 512±25 ms; *P*=0.047 uncorrected paired *t*-test). The response errors also occurred significantly quicker under STN-DBS ON, as quantified by a shorter reaction time in USs (USRT OFF: 586±54 ms; USRT ON: 518±32 ms; *P*=0.024 uncorrected paired *t*-test).

Regarding inhibition performance, STN-DBS increased the SSRT (SSRT OFF: 186±17 ms; SSRT ON: 239±21 ms; *P*=0.014 uncorrected paired *t*-test). STN-DBS also decreased the SSD (SSDOFF: 430±46 ms; SSDON: 348±38 ms; *P*=0.014 uncorrected paired *t*-test). The preparation costs (PC), defined as GFRT–GORT and indicating the proactive inhibition performances,^36^ were not modulated by the stimulation (PCOFF: 84±29 ms; PCON: 94±27 ms; *P*=0.454 uncorrected paired *t*-test). The oddball effect (OE), defined as GCRT-GORT, was not modulated by the stimulation either (OEOFF: 58±13 ms; OEON: 56±13 ms; *P=*0.459). We found no significant difference on these behavioral measurements between the different OCD types (comparing the five washers vs the six checkers, see Table 1).

### Scalp ERPs

**Figure 2:**
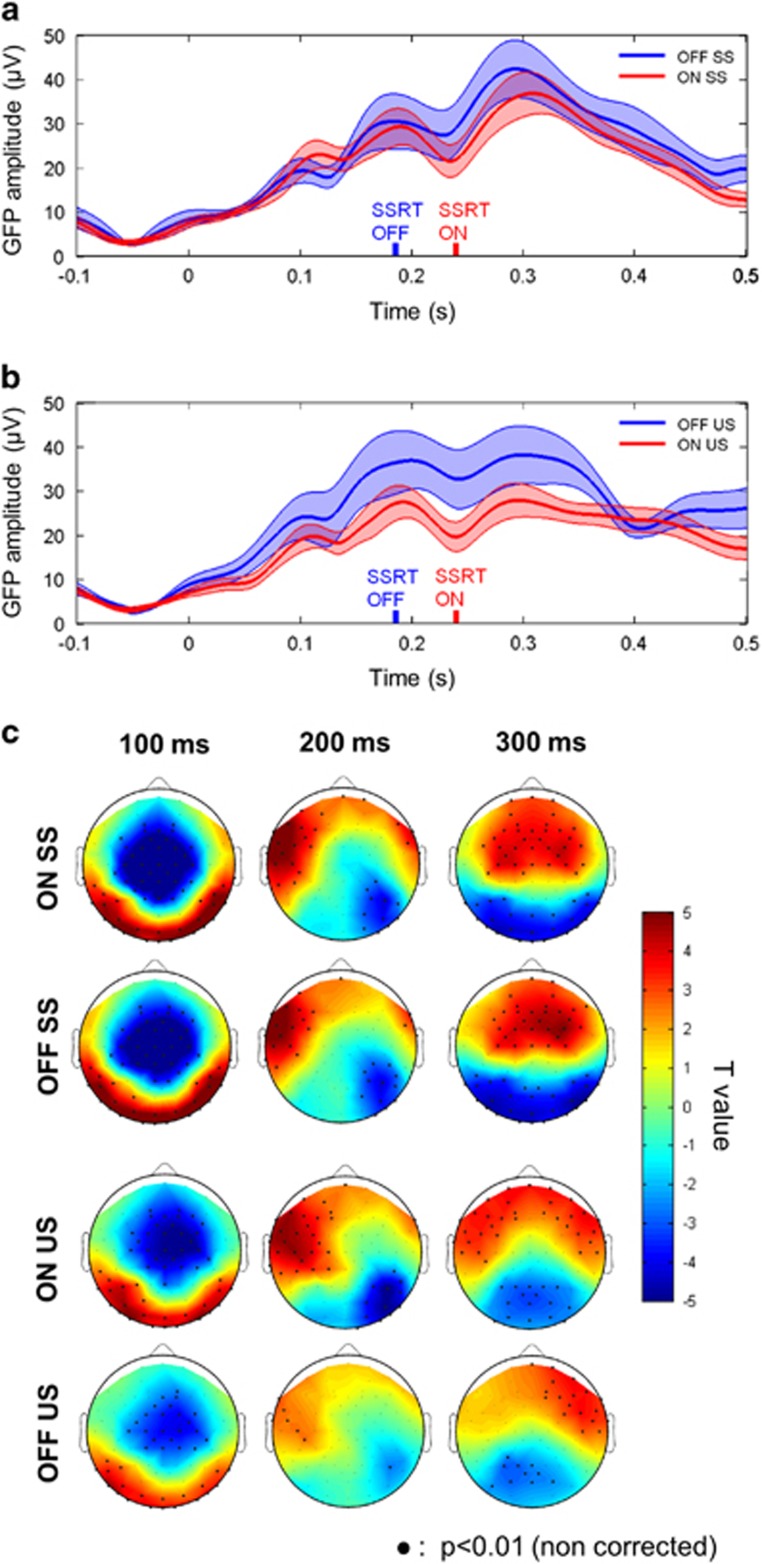
Grand average of the global field power (low-pass-filtered at 10 Hz; ±s.e.m.) for the different conditions: ON stimulation (in red) and OFF stimulation (in blue) (**a**) in the successful stop (SS) trials and (**b**) unsuccessful stop (US) trials. (**c**) Scalp topographies of the data in ON and OFF stimulation conditions for SS and US trials at three latencies: 100 ms (time window: 75-125 ms), 200 ms (time window: 175–225 ms) and 300 ms (time window: 275-325 ms). Significant (0.01 uncorrected) sensors are indicated by dots in bold. GFP, global field power; SSRT, stop signal reaction time.

For both stimulation conditions, the N200 and P300 components of the GFP had lower amplitude in the US than in the SS condition (*P*<0.05 for the ERP on F4), which suggests that these peaks could be linked to inhibition mechanisms (Figure 2). The GFP N200 reached its maximum at similar latencies to the SSRT (from the ERP at F4: N200 mean peak latency OFF DBS=246 ms and ON DBS=235 ms). It was decreased with STN-DBS in both SS and US trials (N200 mean amplitude ON DBS: 18.1 μV^2^ vs OFF DBS: 20.5 μV^2^, *P*=0.007 paired *t*-test across patients). The GFP P300 amplitude was also decreased with STN-DBS in SS and US trials (P300 mean amplitude ON DBS: 35.8 μV^2^ vs OFF DBS: 43.9 μV^2^, *P*=0.035 paired *t*-test across patients). Finally, the GFP P300 latency was significantly delayed by the stimulation in both SS and US trials (mean latency ON DBS: 314 ms vs mean latency OFF DBS: 297 ms, *P*=0.017 paired *t*-test across patients).

Figure 2c shows the statistically significant ERP activity across patients for the four conditions of stimulation and performances, in three windows of interest corresponding to the N100, N200 and P300. The N100 was fronto-central and concomitant to an occipital positive wave. The topographies of the N200 were right lateralized and this postero-central wave was simultaneous to a positive left lateralized frontal wave, which appeared to spread over the right side for the successful stops only. Moreover, at this latency, the amplitude of the signal was statistically significant on fewer electrodes in the OFF condition than in the ON condition. The P300 seemed to be more right lateralized in the OFF condition in both SS and US conditions. It involved slightly more statistically significant electrodes in the right hemisphere in the OFF condition than in the ON condition, where more electrodes from the left hemisphere were activated significantly.

### Source reconstruction

**Figure 3:**
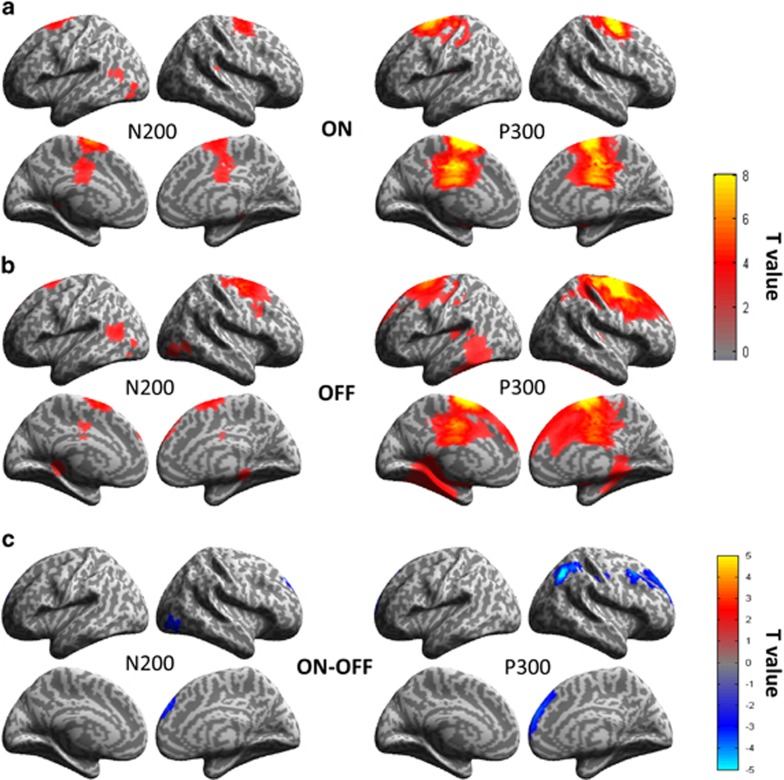
Global network after source reconstruction for successful stop trials in (**a**) ON DBS condition and (**b**) OFF DBS condition. The activated areas common to the 12 patients significantly (*P*<0.005) are reported as T values on the cortical surface on the two windows of interest: centered on the individual N200 and P300 peak latencies. (**c**) Contrast T maps of the stimulation effects (ON–OFF) for the Successful Stop condition. The activated areas modulated by the stimulation for the 12 patients significantly (*P*<0.005) are reported as T values on the cortical surface on the N200 and P300 latencies. The blue areas correspond to stronger activations in OFF than ON stimulation conditions and reciprocally, the red areas correspond to stronger activations in ON than OFF stimulation conditions. DBS, deep brain stimulation.

Group source localization allowed clarifying the network involved in the generation of ERP components N200 and P300 for the two conditions of stimulation for successful stops (Figures 3a and b). At the latencies of the N200, activation was mainly located on the left inferior lateral occipital cortex, the posterior dorsal ACC and the preSMA, as well as on the right superior part of the IFG (in the OFF DBS condition). At the latency of the P300, the activity was stronger in the preSMA and posterior dorsal ACC and extended frontally to the bilateral IFG (on their superior parts) and the DLPFC on the right hemisphere for the OFF DBS condition. There was also activity in the medial inferior and right lateral temporal cortex with DBS OFF.

When comparing ON and OFF DBS conditions for the successful stops (Figure 3c), the only significant effect was a decrease of cortical activity in a limited number of regions. At the latency of the N200, STN-DBS decreased the activity in small parts of the right dorso-medial prefrontal cortex and the right lateral occipital cortex. At the latency of the P300, STN-DBS decreased more significantly the activity in the right hemisphere in the superior lateral parietal cortex, the IFG, the DLPFC and the dorso-medial prefrontal cortex.

### DCM

**Figure 4:**
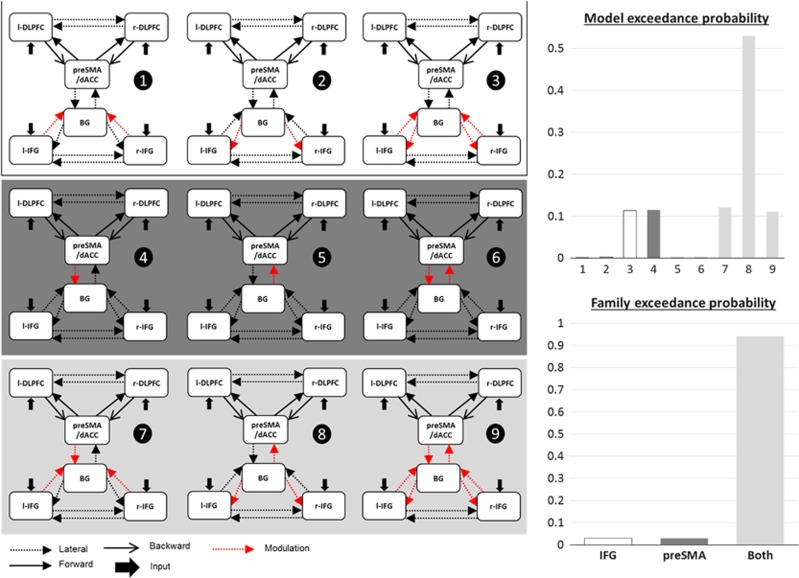
Models tested by dynamic casual modeling on the successful stop trials for both conditions of DBS. The nine tested models were grouped by families depending on the entry to the basal ganglia on which the modulation by DBS was tested: either the inferior frontal gyrus (IFG) or the pre-supplementary motor area (preSMA)/dorsal anterior cingulate cortex (dACC). Bayesian model selection results are displayed for each model and each family of models. BG, basal ganglia; DBS, deep brain stimulation; DLPFC, dorsolateral prefrontal cortex.

The fit of ERPs corresponding to Successful Stop trials by DCM explained 78.2% of the variance (average over all the models and subjects). When comparing the nine models using Bayesian model selection, we found that the modulation of the efferent connections from the BG could explain the data with the highest evidence (49% of exceedance probability). The most probable family of models was the family with modulations of connections with the BG involving both the IFG and preSMA/dACC (Figure 4).

**Figure 5:**
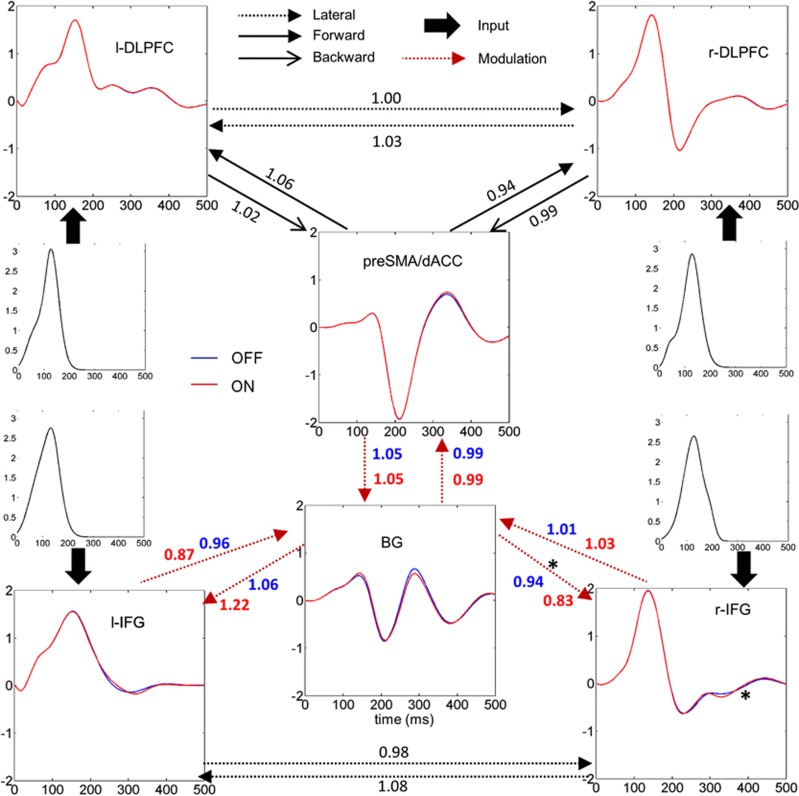
Group averaged dynamic casual modeling time series and effective connectivity on the most probable family (after Bayesian model selection), averaged over the three models of this family. Connectivity weights from the Bayesian model averaging posteriors are indicated on each arrow (exponential of the mean connectivity over all the patients). Asterisk stands for *P<*0.05 in a paired *t*-test between stimulation conditions (on the amplitude time series and connectivity weights). BG, basal ganglia; dACC, dorsal anterior cingulate cortex; DLPFC, dorsolateral prefrontal cortex; IFG, inferior frontal gyrus; preSMA, pre-supplementary motor area.

After Bayesian model averaging, the connection whose modulation by DBS was the most reproducible across patients was the efferent connection from the BG to the right IFG whose strength was decreased with DBS (*P*=0.0181, paired *t*-test). The source time series averaged across models of the wining family were computed (Figure 5). The N200 seemed to be produced mainly by the preSMA/dACC and was not modulated by the stimulation. At the P300 latency, the preSMA/dACC, the BG and the bilateral IFG were the most modulated by the stimulation, but significance was obtained only in the right IFG.

## Discussion

Lesions of the STN were shown to shorten the reaction times to Go signals and to impair the inhibition performance in rats.^37^ We obtained similar effects with STN-DBS: the reaction times and the SSD decreased, whereas the SSRT increased. This finding is consistent with a previous study, where bilateral STN-DBS in Parkinsonian patients induced a shortening of the reaction times in conflict situations.^38^ STN-DBS was also shown to reduce the beta band synchronization, which may control the trade-off between voluntary movement and suppression of prepotent responses, and thereby has an effect on the observed impulsivity increase.^39^ However, because patients with obsessive-compulsive disorder show psychomotor slowing, as reflected in the SST with reaction times longer than in healthy subjects,^40, 41, 42^ it turns out that the observed increase of rapidity in goal-directed behaviors could be beneficial. Because the SST with the tracking method we used keeps the number of errors ~50%, we could not conclude on the effects of STN-DBS on impulsivity.

Patients with OCD are thought to have motor inhibitory performances close to healthy subjects.^15^ Here motor inhibitory performances could be directly quantified from the SSRT:^16^ the shorter the SSRT, the better the performance in inhibition. From SSRT measurements in the different conditions, we showed that inhibition performances of patients with OCD were decreased with STN-DBS, but with no significant effects of STN-DBS on the oddball (conflict effect due to visualization of the rare Stop cue) and on the preparation costs that reflect proactive inhibition (results not shown). Therefore, the alteration of motor inhibition is probably due to an effect of the stimulation on the reactive inhibition alone. The lack of effect of STN-DBS on proactive control has been reported before.^43^ STN-DBS could even improve proactive inhibition in Parkinsonian patients.^19^ In another recent study,^44^ stimulated Parkinsonian patients reacted more automatically to Go cues, but they gained back an appropriate control on their actions in the STOP trials by selectively modulating the proactive inhibition. In the interpretation of the authors of this study, the STN-DBS re-established the executive internal control on the tonic resting state proactive inhibition.

The main ERP correlate of STN-DBS effect on motor inhibition of this study was a reduction of the P300 amplitude and a delay of its latency. In a Go/NoGo study, the NoGo P300 had a lower amplitude in highly impulsive patients,^45^ suggesting that the observed decrease of P300 amplitude could be linked to the increase of impulsivity. This amplitude modulation and the higher delay of those ERPs could explain the decrease of inhibition performances observed when stimulating the STN, the inhibition processes taking longer to complete. The N200 may be associated to the reactive inhibition because its latency is similar to the SSRT. In contrast, the latency of the P300 may be too late to have a causal role on inhibition. The P300 is thus more likely to occur during the maintenance of inhibition, its completion or the monitoring of inhibitory processes.

Functional magnetic resonance imaging studies revealed some regions activated by the Stop processes: the right IFG,^25^ the preSMA/dACC, the parietal cortex^7^ and the DLPFC.^46^ The sources estimated from EEG in our study included some of those areas, with the preSMA/dACC activated at the latency of the SSRT. This result confirmed the implication of these regions in the reactive inhibition network. The DLPFC and IFG were activated later on, suggesting that they are more involved in the maintenance and monitoring of the inhibition than in its initiation. The activity of the inhibition network was decreased by the STN-DBS mainly in the right hemisphere around the DLPFC, the superior part of the IFG and the superior lateral parietal cortex. Because the DLPFC was shown to be hyperactive in patients with OCD in a task requiring cognitive control,^47^ one can speculate that a mode of action of STN-DBS to decrease obsessive-compulsive symptoms could be the reduction of the feedback control of frontal areas, such as the DLPFC. Given that deficits in goal-directed control have been proposed to confer vulnerability for developing rigid habits,^48^the STN-DBS reduction of the cognitive control of frontal areas could in turn allow patients with OCD to engage more in goal-directed behaviors, thereby leading to a positive effect of STN-DBS on the compulsion aspects of OCD symptoms.

We used DCM to model the change of effective connectivity with STN-DBS, using a simplified network including 6 regions of interest and 14 connections, according to the main assumptions of the models developed in Wiecki and Frank,^26^Chambers *et al.*,^49^ and Aron.^50^ We found that the more reproducible effects on inhibition network of STN-DBS may be a modulation of efferent connections from the BG to the cortex. Although DCM for EEG is not precise enough to clearly disentangle the biophysical mechanisms of action of DBS, our findings add some evidence that DBS could modulate specifically orthodromic axons from the STN up to the thalamus output towards cortical areas. Disruption of the STN activity by DBS could explain the modulation of these efferent connections. The connection with the more reproducible modulation across patients was the connection from the BG to the right IFG. This region is a key node of inhibition networks, which could explain the observed decrease of performance with DBS.

To the best of our knowledge, this work was the first assessment of the impact of STN-DBS in patients with OCD using EEG. We showed that STN-DBS had distributed effects on the network of motor inhibition, with the main effect to decrease inhibition-related responses. These new findings are very interesting to understand the neuronal correlates of motor inhibition, but cannot fully explain the clinical response to STN-DBS. To address this issue, it would be relevant in the future to apply similar methods in tasks involving obsessive-compulsive symptoms provocations or inhibition and control of intrusive thoughts. Such studies could probably relate modulation of connectivity to the reduction of symptoms by DBS, and therefore would help to better understand the pathophysiology of obsessive-compulsive disorders.

## Figures and Tables

**Table 1:** Clinical and demographic information on patients

*Patient number*	*Gender*	*Age (years)*	*Age at surgery (years)*	*Duration of the disease before surgery (years)*	*Duration of the DBS (months)*	*OCD subtype*	*YBOCS pre-surgery*	*Treatment at the time of the study*
1	M	46	39	18	71	Checker	37	Fluvoxamine 200 mg per day Lorazepam 4 mg per day
2	F	49	42	25	64	Washer	30	Aripiprazole 30 mg per day Olanzapine 5 mg per day Escitalopram 20 mg per day Clomipramine 75 mg per day
3	M	39	36	17	32	Washer/ordering	32	Paroxetine 60 mg per day
4	F	53	49	39	51	Checker	35	Fluoxetine 20 mg per day Clomipramine 25 mg per day
5	M	37	34	13	22	Checker/repeater	32	Clomipramine 150 mg per day Oxazepam 175 mg per day Alimemazine 50 mg per day
6	F	41	38	11	35	Washer	36	None
7	F	43	40	15	32	Washer	36	Fluvoxamine 200 mg per day Hydroxyzine 50 mg per day Clomipramine 25 mg per day
8	F	41	37	5	44	Checker	32	Venlafaxine 37.5 mg per day Clotiazepam 1.5 mg per day
9	M	30	27	10	25	Checker/washer	38	Sertraline 50 mg per day Aripiprazole 20 mg per day Methyphenidate 60 mg per day Pitolisant 20 mg per day
10	F	56	52	25	51	Washer	40	Zopiclone 7.5 mg per day Aripiprazole 2.5 mg per day Hydroxyzine 100 mg per day
11	F	33	33	21	5	Checker	30	Venlafaxine 150 mg per day
12	F	33	33	26	25	Checker	34	Fluoxetine 20 mg per day Levothyroxine 125 μg per day

Abbreviations: DBS, deep brain stimulation; F, female; M, male; OCD, obsessive-compulsive disorder; YBOCS, Yale-Brown Obsessive Compulsive Scale.
