# Supplementary material for: Modulation of motor inhibition by subthalamic stimulation in obsessive-compulsive disorder
Source: Transl Psychiatry. 2016 Oct 18;6(10):e922–. doi: 10.1038/tp.2016.192 (PMC5315551; doi:10.1038/tp.2016.192)
Supplement: Supplementary Information [file tp2016192x1.docx]

**Modulation of motor inhibition by subthalamic stimulation in obsessive-compulsive disorder**

Astrid Kibleur PhD ^1,2^, Guillaume Gras-Combe MSc ^1,2,+^, Damien Benis PhD ^1,2,++^, Julien Bastin PhD ^1,2^, Thierry Bougerol MD PhD ^1,2,3^, Stéphan Chabardès MD ^1,2,4^, Mircea Polosan MD PhD ^1,2,3^, Olivier David PhD ^1,2,*^

^1^ Univ. Grenoble Alpes, F-38000 Grenoble, France

^2^ Inserm, U1216, Grenoble Institut des Neurosciences, F-38000 Grenoble, France

^3^ Clinique Universitaire de Psychiatrie, Pôle Psychiatrie Neurologie, Centre Hospitalier Universitaire, F-38000 Grenoble, France

^4^ Clinique Universitaire de Neurochirurgie, Pôle Tête et Cou, Centre Hospitalier Universitaire, F-38000 Grenoble, France

* Corresponding author:

Olivier David, PhD

Grenoble Institut des Neurosciences – Chemin Fortuné Ferrini – Bât EJ Safra – CHU

38700 La Tronche, France

Email: [Olivier.David@inserm.fr](mailto:oOlivier.Ddavid@insermujf-grenoble.fr)

Tel: +33 4 56 52 05 86

Fax: +33 4 56 52 05 98

^+^ Present address: Département de Neurochirurgie, CHU Montpellier, 80 avenue Auguste Fliche, 34295 Montpellier Cedex 5, France

^++^ Present address: Université de Genève / FPSE, UNIMAIL, 40 Boulevard du Pont-d'Arve, 1211 Genève, Suisse

1. *Stop Signal Reaction Time computation*

As required by the horse-race model ^1^, we assumed that the SSRT was constant for each patient. To compute its value, we used the n^th^ Go reaction time (Go RT) method, instead of the mean Go RT as classically done ^1^, in order to take into account the fact that the success rate in the STOP trials (%SS) was not exactly 50%. We sorted the Go RTs (from the GO trials) in an increasing order and determined the n^th^ Go RT (GORT_n_) that separated the trials into the fast Go (FG) and the slow Go (SG) with the constraint $\%SG=\%SS$. To estimate the SSRT, the patient’s critical SSD (SSD_c_) was simply subtracted from GORT_n_ ^2^:

. (1)

The SSD_c_ was estimated from the inhibition function $W\left(SSD\right)$, which is a sigmoid curve representing the cumulative probability of response times in the STOP trials when the SSD changes ^3^. We used the Weibull function that best fitted the inhibition function (in the least square sense) to retrieve the SSD_c_ ^4^, the proportion of failed stops ($\%US$) in the STOP trials being equal to $W\left(SSD_{c}\right)$:


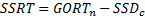
 (2)

where γ is the maximum of the inhibition function (its value ranged between 0.9 and 1 depending on the patient), δ is the minimum of the inhibition function (between 0 and 0.1), α is the time at which the inhibition function reaches 64% of γ and β is the slope of the inhibition function.

1. *Estimation of motor inhibition ERPs*

By definition, the Stop trial is successful (SS) when the sum of SSD and SSRT is shorter than the Go RT. We could thus assume that Go processes involved during SS trials were slow and their EEG component could be estimated from GO trials with the slowest reaction times, *i.e.* the slow GO (SG) trials. In the same line, when the sum of SSD and SSRT is longer than the Go RT, the Stop trial is unsuccessful (US). Because the EEG correlates of fast GO (FG) processes could be estimated from GO trials with the shortest reaction times, we removed the GO component from the EEG evoked responses to the US trials using the FG trials and to the SS using the SG trials.

Our implementation of the estimation of FG and SG responses improved the procedure proposed in ^5,6^. Instead of using a binary classification of FG and SG trials to estimate the fast and slow GO EEG evoked responses, we simply used a weighted average across GO trials according to the GORT (Figure 1). The weighting kernel was composed of two sigmoids that were designed to diminish the contribution of trials at the limit between FG and SG trials and of trials with abnormal responses (RT below GORT_min_ or above GORT_max_). The weighted average can be summarized as

$\begin{matrix} \left\langle FG\right\rangle=\left\langle\frac{GO_{i}}{\left(1+\exp\left(-0.5\times\left(GORT_{i}-GORT_{\min}\right)\right)\right)\left(1+\exp\left(0.05\times\left(GORT_{i}-GORT_{n}\right)\right)\right)}\right\rangle_{i} \\ \left\langle SG\right\rangle=\left\langle\frac{GO_{i}}{\left(1+\exp\left(0.5\times\left(GORT_{i}-GORT_{\min}\right)\right)\right)\left(1+\exp\left(-0.05\times\left(GORT_{i}-GORT_{n}\right)\right)\right)}\right\rangle_{i} \end{matrix}$ (3)

where *GO_i_* is the EEG response to trial *i*.

Under the assumption of independence of the ERP components of GO and STOP processes, one can estimate the EEG response to the inhibitory process during SS or US trials by subtracting the previously inferred EEG response in the SG or FG trials, respectively. To do so, the ERPs to STOP trials were centred on the stop signal cue (SSC) because the inhibition is time locked to this stimulus. This was easily done for US and SS trials, but in FG and SG trials the stop signal cue did not exist. We thus assigned a virtual stop signal cue (VSSC) to GO trials positioned SSD ms after the GO cue, using the mean SSD value of US trials for FG trials and the mean SSD value of SS trials for SG trials. Finally, the evoked response to the inhibition process from each Stop trial was computed as

$\begin{matrix} US_{corrected}=US_{centred\_on\_SSC}-\left\langle FG\right\rangle_{centred\_on\_VSSC} \\ SS_{corrected}=SS_{centred\_on\_SSC}-\left\langle SG\right\rangle_{centred\_on\_VSSC} \end{matrix}$. (4)

**References**

1 Logan GD, Cowan WB. On the ability to inhibit thought and action: A theory of an act of control. *Psychological Review* 1984; **91**: 295.

2 Band GPH, van der Molen MW, Logan GD. Horse-race model simulations of the stop-signal procedure. *Acta Psychol (Amst)* 2003; **112**: 105–142.

3 Verbruggen F, Logan GD. Models of response inhibition in the stop-signal and stop-change paradigms. *Neurosci Biobehav Rev* 2009; **33**: 647–661.

4 Hanes DP, Patterson WF, Schall JD. Role of frontal eye fields in countermanding saccades: visual, movement, and fixation activity. *J Neurophysiol* 1998; **79**: 817–834.

5 Kok A, Ramautar JR, De Ruiter MB, Band GPH, Ridderinkhof KR. ERP components associated with successful and unsuccessful stopping in a stop-signal task. *Psychophysiology* 2004; **41**: 9–20.

6 Ramautar JR, Kok A, Ridderinkhof KR. Effects of stop-signal modality on the N2/P3 complex elicited in the stop-signal paradigm. *Biol Psychol* 2006; **72**: 96–109.


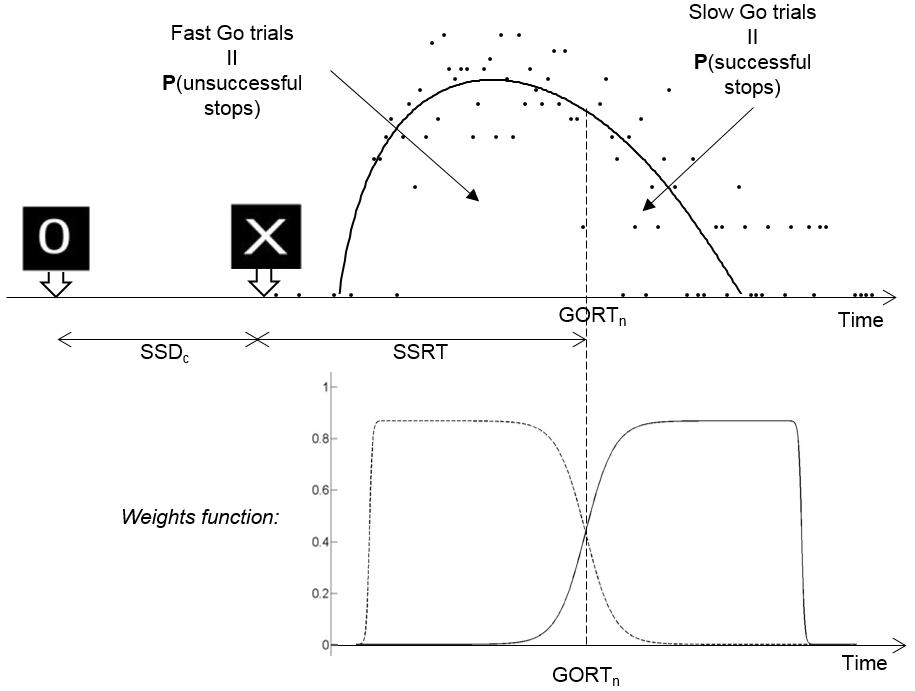


**Figure 1:** The Go reaction time density function (example from one session). The fast Go trials where the subject would have failed to inhibit if a Stop signal had occurred, are dissociated from the slow Go trials where the subject would have successfully stopped. Fast Go and Slow Go signals (for the correction of the unsuccessful Stop trials and the successful Stop trials respectively), are computed by applying 2 weighted averages on both types of Go trials (fast and slow).
